# Cohesive strength of nanocrystalline ZnO:Ga thin films deposited at room temperature

**DOI:** 10.1186/1556-276X-6-309

**Published:** 2011-04-07

**Authors:** Anura Priyajith Samantilleke, Luís Manuel Fernandes Rebouta, Vitor Garim, Luis Rubio-Peña, Senetxu Lanceros-Mendez, Pedro Alpuim, Sandra Carvalho, Alexey V Kudrin, Yury A Danilov

**Affiliations:** 1 Centro de Física, Universidade do Minho, Azurém, 4800-058 Guimarães, Portugal; 2 Engineering School, University of Cadiz, C/Chile, 1. 11002 Cádiz, Spain; 3 Physical-Technical Research Institute, N. I. Lobachevskiy State University, Nihzniy Novgorod, Russia

## Abstract

In this study, transparent conducting nanocrystalline ZnO:Ga (GZO) films were deposited by dc magnetron sputtering at room temperature on polymers (and glass for comparison). Electrical resistivities of 8.8 × 10^-4 ^and 2.2 × 10^-3 ^Ω cm were obtained for films deposited on glass and polymers, respectively. The crack onset strain (COS) and the cohesive strength of the coatings were investigated by means of tensile testing. The COS is similar for different GZO coatings and occurs for nominal strains approx. 1%. The cohesive strength of coatings, which was evaluated from the initial part of the crack density evolution, was found to be between 1.3 and 1.4 GPa. For these calculations, a Young's modulus of 112 GPa was used, evaluated by nanoindentation.

## Introduction

Doped ZnO thin films are widely used as transparent electrodes in optoelectronic and electro-optic devices such as solar cells and flat panel displays [1-3], because of their unique properties, specifically low electrical resistivity and high transmittance in the visible spectral region [4]. These properties are obtained using substrate temperatures higher than 200°C, but growing interest in flexible substrates has led to the use of polymeric alternatives, which require the deposition of films at low temperature [5]. Furthermore, the deposition on polymeric substrates decreases the quality of the film properties [6]; therefore, the pursuit toward an understanding of the structural, electromechanical and electro-optical properties of nanocrystalline (nc) thin films is crucial for device applications.

## Experimental details

ZnO:Ga (GZO) thin films were deposited by dc-magnetron sputtering on glass and polyethylene naphthalate (PEN) substrates, under an Ar atmosphere with a base pressure of 2 × 10^-4 ^Pa, from a GZO target (zinc oxide/gallium oxide, 95.5/4.5 wt.%) of 2" diameter. A target current density of 0.6 mA/cm^2 ^was applied, and a deposition rate of 21 nm/min was obtained. No bias was applied to the substrate holder during the depositions, which took place at room temperature. The working pressure (*P*_w_) was varied from 0.41 to 0.86 Pa, with the target-to-substrate distance kept at a constant 8 cm. The crystallinity and crystal orientation was studied using a Bruker AXS Discover D8 (Madison, USA) for X-ray diffraction (XRD). Glass substrates were used to avoid the presence of polymer substrate peaks. The electrical resistivity, carrier concentration and Hall mobility of the coatings on glass substrates were all measured using Van der Pauw geometry under a magnetic field of 1 Tesla. The electromechanical tests were carried out on 10 × 40 mm^2 ^samples using a computer-controlled tensile testing machine (Minimat, Polymer Labs, Loughborough, UK), which was mounted on an optical microscope stage (Nikon Optiphot-100, Tokyo, Japan). One of the grips of the instrument was displaced at a constant speed of 0.2 mm/min. The applied load and stage displacement values were recorded at 1-s intervals. Crack development was recorded through a CCD camera connected to the microscope, with the evolution of the crack density obtained by the subsequent video analysis. The thickness of the polymer substrates was measured using a Fischer Dualscope MP0R instrument (Sindelfingen, Germany).

## Results and discussion

### Structural characterization

**Figure 1:**
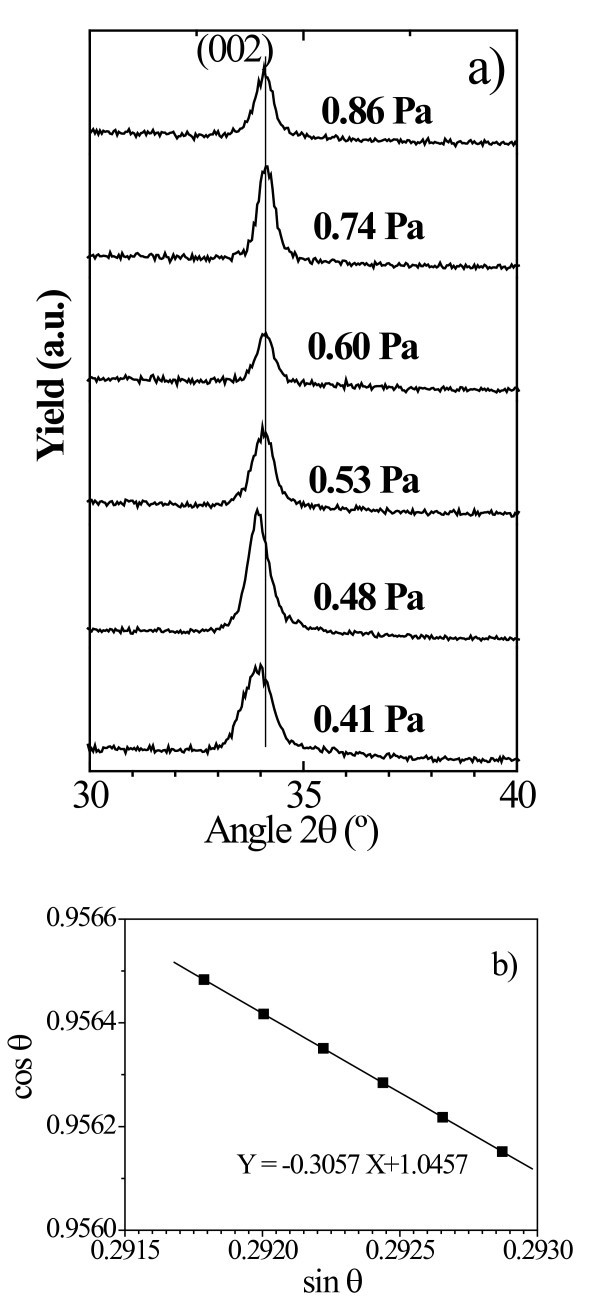
**XRD analysis for GZO thin films prepared under different *P*_w_s**.

Figure 1a shows the XRD spectra obtained for nc GZO thin films (approx. 100-nm thick) as a function of the *P*_w_, where only the ZnO (002) peak, at approx. 34°, is observed. The spectra reveal a highly textured hexagonal phase with a wurtzite structure. A lower *P*_w _resulted in samples with a higher c-lattice parameter. In the thin films prepared with a *P*_w_, between 0.41 and 0.86 Pa, the (002) peak position shifted from 2θ = 33.93° (*c *= 0.528 nm) to 2θ = 34.06° (*c *= 0.525 nm). The full-width at half-maximum (FWHM) can be expressed as a linear combination of the lattice strain and crystalline size. The effects of strain and particle size on the FWHM can be expressed as [7](1)

where β is the measured FWHM, θ is the Bragg angle of the peak, λ is the X-ray wavelength (1.5418 Å), ε is the effective particle size and τ is the effective strain. The average particle size, calculated from the plot cos θ versus sin θ shown in Figure 1b, was 8.7 nm. The particle size (*D*_v_) calculated from Scherrer's formula (*D*_v _= 0.94λ/(β cos θ)), was 8.9 nm, which is very close to that calculated from Equation 1 [8]). The presence of strain in the ZnO crystal lattice, caused indirectly by *P*_w, _can be expected to exert significant influence on the mechanical properties of the nc-GZO thin film.

### Optical properties

**Figure 2:**
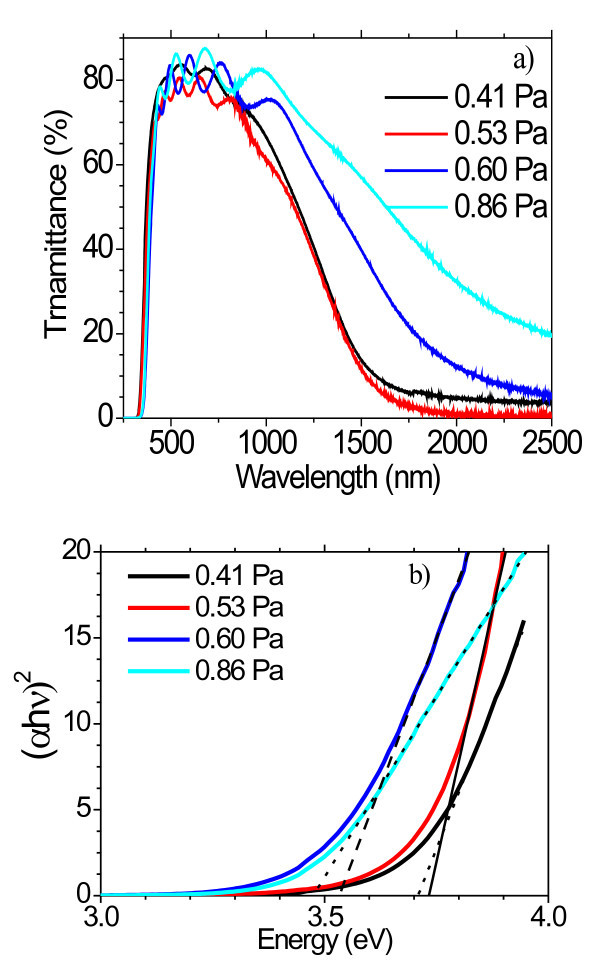
**Optical transmittance of GZO/glass at various *P*_w_s**.

The nc nature of the thin films influences both optical and electrical performance. Figure 2 shows optical transmittance as a function of wavelength for thick GZO films (approx. 700 nm) prepared on glass at various *P*_w_, using air as a reference. The near infra-red transmittance is lower for *P*_w _values of 0.41 and 0.53 Pa and increases with higher *P*_w_, which is consistent with the changes observed in the electrical resistivity (discussed in the next section). The optical band gap for GZO films was calculated by plotting (α*hν*)^2 ^as a function of photon energy (*hν*) and extrapolating the linear region of (α*hν*)^2 ^to energy axis where (α*hν*)^2 ^corresponds to zero. Figure 2b shows the plot of (α*hν*)^2 ^as a function of photon energy (*hν*) for GZO films. From these plots, it can be seen that the value of the bandgap of GZO decreased from 3.73 eV (0.41 Pa) to 3.48 eV (0.86 Pa), which can be understood in the context of the Burstein Moss shift [9].

### Electrical properties

**Figure 3:**
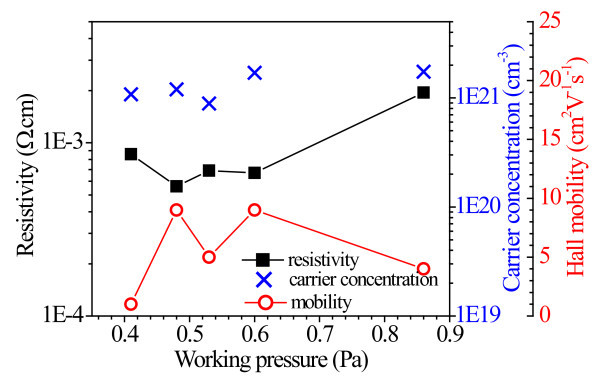
**The electrical resistivity, carrier concentration and Hall mobility for GZO/glass as a function of the *****P***_**w**_.

The electrical resistivity, charge carrier concentration and Hall mobility as a function of the *P*_w_, for GZO films deposited on glass, are shown in Figure 3. The resistivity of GZO samples decreased initially, and then increased with the *P*_w_. In general, the average resistivity was low (approx. 10^-4 ^Ω cm), which can be attributed to high carrier concentration. Considering the similarity in the conduction mechanism of electrons in GZO and ITO, the grain boundary (GB) and ionized impurity scattering processes can be considered the two dominant mechanisms, limiting electron transport in nc-GZO films, as in the case of ITO, where other scattering mechanisms such as lattice vibrations and neutral impurity scattering may typically be neglected [10]. The relative importance of the scattering mechanism is dependent on film quality and carrier concentration. Unlike intrinsic ZnO, where the conduction is generally controlled by GB-scattering, in doped ZnO at high electron density (>10^20 ^cm^-3^), the ionized impurity scattering can be expected to dominate, which explains the low values of electron mobility (<10 cm^2^V/s) [11].

### Tensile tests

**Figure 4:**
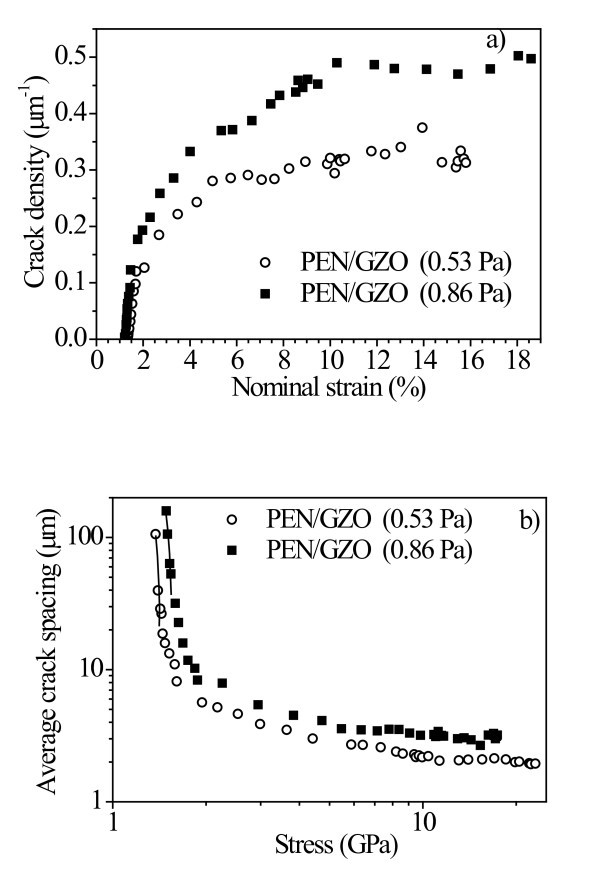
**Cracking density as a function of the substrate nominal strain for different GZO coatings deposited on PEN (82 μm) and the crack density evolution of the PEN/GZO coatings**.

Tensile tests were performed at a constant strain rate on PEN substrates (82 μm) coated with GZO films (approx. 100 nm) prepared under two different *P*_w_s to increase nominal strains. The PEN substrate is isotropic, and the elastic modulus was 4.23 GPa, as measured through the tensile test on uncoated substrate. The cracking densities as a function of the substrate nominal strain for two different GZO coatings (0.53 and 0.86 Pa) are shown in Figure 4a. The crack densities at saturation of these two PEN/GZO films were 0.316 and 0.515 μm^-1^, respectively. The coatings have similar properties and thicknesses, with small differences causing variations wholly within acceptable margins of error. Using the weakest link model, the coating's cohesive strength was evaluated from the early stages of the fragmentation process, assuming a Weibull-type, size-dependent probability of failure for the coating fragments of length *ℓ *under a stress σ [12,13]:(2)

Assuming that the residual stresses were negligible, in the initial stage of fragmentation, the average fragment length was related to the stress acting in the coating. The average fragment length (*ℓ*) is *ℓ*_0_(σ/β)^-α^, where a normalizing factor (*ℓ*_0_) of 1 μm was chosen. In addition, σ is the axial stress acting in the coating, and α and β are the Weibull shape and scale parameters, respectively. These parameters were derived from a plot of ln(*ℓ*) versus ln(σ), shown in Figure 4b, using the initial part of the crack density evolution of the PEN/GZO coatings, displayed in Figure 4a.

The cohesive strength of the coating at critical length (***ℓ***_c_) can be expressed as(3)

where Γ is the gamma function, *ℓ*_c _= (3/2)*ℓ*_sat _is the critical length and *ℓ*_sat _is the experimental mean fragment length at saturation, which is also the inverse of the crack density at saturation [14]. As shown in Figure 4a, the GZO coatings prepared at *P*_w _of 0.53 and 0.86 Pa revealed mean fragment lengths at saturation of 3.11 and 1.94 μm, respectively.

In order to take into account its influence, the internal stress was evaluated, and the COS and the coating strength obtained with this method were corrected.(4)

where σ_i _is the internal stress and ε*_i_*= σ*_i_*(1 - ν_c_)/*E*_c_, the internal strain, with *E*_c _and ν_c _being the Young's modulus and Poisson ratio, respectively, of the coating. Young's modulus of GZO was measured by nanoindentation at 113 and 112 GPa from samples prepared at 0.60 and 0.86 Pa, respectively. Young's modulus of the PEN substrate was determined from tensile testing (4.23 GPa). The cohesive strength of the coatings, which was evaluated from the initial part of the crack density evolution, was found to be between 1.3 and 1.4 GPa. The crack onset strains (COS^cor^) occurs for nominal strains of 1.1 and 1.0%, respectively. The COS and cohesive strength of GZO are relatively similar to those reported in the literature for other polycrystalline conducting films [15].

## Summary

The material, opto-electrical properties, COS, the coating cohesive strength, as well as the influence of mechanical strain on the electrical properties of nc GZO thin films were investigated. The estimated average crystalline size of nc-GZO films was approx. 8.7 nm, and the bandgap shifted from 3.73 eV (0.41 Pa) to 3.48 eV (0.86 Pa), where the low resistivity (approx. 10^-4 ^Ω cm) and the high electron density (>10^20 ^cm^-3^) explain the dominating scattering process as the ionized impurity scattering. The COS is similar for different GZO coatings and occurs for nominal strains approx. 1%. The cohesive strength of coatings, which was evaluated from the initial part of the crack density evolution, was found to be between 1.3 and 1.4 GPa, while the Young's modulus was evaluated by nanoindentation.

## Abbreviations

COS: crack onset strains; FWHM: full-width at half-maximum; GB: grain boundary; nc: nanocrystalline; PEN: polyethylene naphthalate; XRD: X-ray diffraction.

## Competing interests

The authors declare that they have no competing interests.

## Authors' contributions

LR and SLM proposed the research work, and with APS coordinated the collaborations and carried out the analysis and interpretation of the experimental results. VG and LRP participated in sample processing, electromechanical experimental measurements, and analysis and interpretation of the results. PA, AVK and YAD carried out electrical measurements and SC performed the nanoindentation measurements. All authors read and approved the final manuscript.

## References

[B1] Fonrodona M Escarré J Villar F Soler D Asensi JM Bertomeu J Andreu J PEN as substrate for new solar cell technologies Sol Energy Mater Sol Cells 2005 89 37 10.1016/j.solmat.2004.12.006

[B2] Kyaw AKK Sun XW Zhao JL Wang JX Zhao DW Wei XF Liu XW Demir HV Wu T Top-illuminated dye-sensitized solar cells with a room-temperature-processed ZnO photoanode on metal substrates and a Pt-coated Ga-doped ZnO counter electrode J Appl Phys D Appl Phys 2011 44 045102 10.1088/0022-3727/44/4/045102

[B3] Taylor MP Readey DW van Hest MFAM Teplin CW Alleman JL Dabney MS Gedvilas LM Keyes BM To B Perkins JD Ginley DS The Remarkable Thermal Stability of Amorphous In-Zn-O Transparent Conductors Adv Funct Mater 2008 18 3169 10.1002/adfm.200700604

[B4] Hamberg I Granqvist CG Evaporated Sn-doped In2O3 films: Basic optical properties and applications to energy-efficient windows J Appl Phys 1986 60 R123 10.1063/1.337534

[B5] Fortunato E Gonçalves A Assunção V Marques A Águas H Pereira L Ferreira I Martins R Growth of ZnO:Ga thin films at room temperature on polymeric substrates: thickness dependence Thin Solid Films 2003 442 121 10.1016/S0040-6090(03)00958-1

[B6] Lewis BG Paine DC Applications and Processing of Transparent Conducting Oxides MRS Bull 2000 25 22 10.1557/mrs2000.147

[B7] Gu F Wang SF Lu MK Zhou GJ Xu D Yuan DR Structure Evaluation and Highly Enhanced Luminescence of Dy3+-Doped ZnO Nanocrystals by Li+ Doping via Combustion Method Langmuir 2004 20 3528 10.1021/la049874f 15875379

[B8] Cullity BD Stock SR Elements of X-Ray Diffraction 2001 3 NJ: Prentice-Hall Inc 167 171 ISBN 0-201-61091-4

[B9] Park JB Park SH Song PK Electrical and structural properties of In-doped ZnO films deposited by RF superimposed DC magnetron sputtering system J Phys Chem Solids 2010 71 669 10.1016/j.jpcs.2009.12.062

[B10] Robbins JJ Harvey J Leaf J Fry C Wolden CA Transport phenomena in high performance nanocrystalline ZnO:Ga films deposited by plasma-enhanced chemical vapor deposition Thin Solid Films 2005 473 35 10.1016/j.tsf.2004.06.154

[B11] Minami T Transport phenomena in high performance nanocrystalline ZnO:Ga films deposited by plasma-enhanced chemical vapor deposition MRS Bull 2000 25 38 10.1557/mrs2000.149

[B12] Weibull W A statistical distribution function of wide applicability J Appl Mech 1951 18 293

[B13] Leterrier Y Boogh L Andersons J Månson J-AE Adhesion of silicon oxide layers on poly(ethylene terephthalate). I: Effect of substrate properties on coating's fragmentation process J Polym Sci B Polym Phys 1997 35 1449 10.1002/(SICI)1099-0488(19970715)35:9<1449::AID-POLB15>3.0.CO;2-6

[B14] Leterrier Y Durability of nanosized oxygen-barrier coatings on polymers Prog Mater Sci 2003 48 1 10.1016/S0079-6425(02)00002-6

[B15] Leterrier Y Médico L Demarco F Månson J-AE Betz U Escola MF Olsson MK Atamny F Mechanical integrity of transparent conductive oxide films for flexible polymer-based displays Thin Solid Films 2004 460 156 10.1016/j.tsf.2004.01.052

